# Inhibition effect of AGEs formation *in vitro* by the two novel peptides EDYGA and DLLCIC derived from *Pelodiscus sinensis*

**DOI:** 10.3389/fnut.2025.1537338

**Published:** 2025-01-30

**Authors:** Nuo Chen, Nan Wang, Qiaoyun Fang, Zuolong Yu, Yiyuan Hu, Jiancang Jin, Shengli Yang

**Affiliations:** ^1^The College of Pharmaceutical Science, Zhejiang University of Technology, Hangzhou, China; ^2^College of Biology and Environmental Engineering, Zhejiang Shuren University, Hangzhou, China

**Keywords:** peptides, inhibition, AGEs, molecular docking, antiglycation

## Abstract

The antioxidant activity of natural products is closely related to their antiglycation effects. This study aimed to examine the antiglycation activity and elucidate the underlying mechanisms of two specific peptides, EDYGA (Glu-Asp-Tyr-Gly-Ala) and DLLCIC (Asp-Leu-Leu-Cys-Ile-Val), derived from protein hydrolysates of the *Pelodiscus sinensis*. Both EDYGA and DLLCIC were efficient in bovine serum albumin (BSA)/glucose model to inhibit BSA glycation, while DLLCIC showed higher antiglycation activity than EDYGA. Firstly, it was found that EDYGA and DLLCIC could inhibit the formation of NEG and AGEs. Moreover, EDYGA and DLLCIC were able to maintain the protein secondary structure and stabilize the band positions (amide I & II). Additionally, molecular simulations indicated that DLLCIC can spontaneously interact with the central site of BSA, specifically at Lys114 and Glu424 residues, through hydrogen bonds with an energy strength of −0.7 kcal/mol. Furthermore, CCK-8 and morphological experiments confirmed that EDYGA and DLLCIC improved cell survival against AGEs-induced cytotoxicity, with EC50 values of 17.64 μM for EDYGA and 15.08 μM for DLLCIC. These findings serve as a significant reference for the development of EDYGA and DLLCIC as effective antiglycation agents in the prevention of glycation-mediated diseases.

## 1 Introduction

Glycation is a reaction between reducing sugars and the free amino residues of proteins without enzyme intervention (1, 2). This reaction involves a series of intermediates, such as Schiff bases and Amadori products, which play a pivotal role in the formation of advanced glycation end products (AGEs) (3, 4). AGEs are the end products of glycation reaction, which are characterized due to their high reactivity, cross-linking, and fluorescence properties (1). Glycation-induced structural and functional changes in proteins have been identified as a major link between diabetes and its severe complications such as retinopathy, neuropathy, and cardiovascular complications (5). To control related diseases caused by glycosylation, treatment methods can be developed based on antiglycosylation mechanisms. Due to the complexity of the reaction, different approaches have been proposed to inhibit the glycation and formation of advanced glycation end products such as (a) carbonyl scavenging (b) inhibition of early glycation reaction (c) inhibition of advanced glycation reaction (d) masking of lysine residues in proteins and (e) protecting the native protein conformation. Some synthetic compounds have antiglycation activity such as aminoguanidine (AG). However, the synthesized compounds are highly toxic (6, 7).

In recent years, the inhibition of the formation of AGEs by natural products has aroused considerable interest, which will be a research hot topic (5). Currently, several natural compounds with antioxidant properties show good inhibitory activity against AGE formation and appear to have minimal toxic effects. The natural compounds that potentially inhibit the formation of AGEs are divided into the following six classes based on their structural properties: polyphenols, polysaccharides, terpenoids, vitamins, alkaloids, and peptides (8, 9). As previously reported in the literature, bioactive peptides exhibited excellent inhibitory effects on protein glycation. Kuerban et al. (10) found that the *Lens culinaris* hydrolysis peptides could inhibit AGEs formation in fructose (15 mmol/L)-bovine serum albumin (BSA) system. Kuerban et al. (11) reported that the *Vicia faba* peptides fractions (less than 3 ku) could capture extra fructose and glucose, with *in vitro* inhibit AGEs formation. Moreover, Aydın et al. (12) observed that carnosine prevents oxidative stress and AGE formation in a D-galactose-induced aging rat model through its antioxidant and antiglycation properties. Additionally, glutathione, an important source of reducing power in the body, prevents glycation by glucose more effectively than carnosine (13, 14). Han et al. (15) observed that a dipeptide, Asn-Trp, from computer-aided simulation of *yam dioscorin* hydrolysis presented strong antiglycation activity and exhibited protection against methylglyoxal-induced cell apoptosis.

Research on small-molecule antioxidants has attracted considerable attention in recent years. These compounds play a crucial role in slowing down the aging process and preventing chronic diseases by scavenging free radicals and reducing oxidative stress. Among them, polyphenols, including tea polyphenols (16), flavonoids (17), and anthocyanins (18), are among the most extensively studied due to their potent antioxidant properties. These bioactive compounds are abundant in plants and are known for their ability to neutralize oxidative damage. Alongside polyphenols, other natural antioxidants such as vitamin C, vitamin E (19), and glutathione (20) also exhibit significant antioxidant effects. In addition, synthetic small-molecule antioxidants like butylated hydroxyanisole and butylated hydroxytoluene (21) have found applications in the food industry. Small-molecule antioxidant peptides, derived from natural proteins via hydrolysis, are increasingly recognized for their strong antioxidant properties and safety. They are easily absorbed by the body and work through mechanisms like metal ion chelation, free radical scavenging, and lipid peroxidation inhibition (22). Studying small-molecule antioxidant peptides is crucial for creating natural and safe antioxidants for food and pharmaceutical use. Antioxidant and antiglycation mechanisms both suppress free radicals and regulate oxidative stress. This stress not only generates free radicals but also speeds up glycation, resulting in AGEs accumulation. AGEs trigger more oxidative damage, creating a cycle. Antioxidants help by neutralizing free radicals and preventing glycation, thus reducing AGEs. This suggests that combining antioxidants and antiglycation approaches could effectively combat aging and chronic diseases.

The BSA/glucose system is a common model for studying AGEs by the reaction of the carbonyl group of glucose with the free amino group of the protein. Previous studies have shown that free radicals are involved in the formation of AGEs (23, 24). In addition, it has been reported that antioxidants and radical scavengers inhibit these processes (25, 26). EDYGA (Glu-Asp-Tyr-Gly-Ala) and DLLCIC (Asp-Leu-Leu-Cys-Ile-Val) are derived from *Pelodiscus sinensis*, which are potent ARE-luciferase inducers with antioxidant properties (27). The structure of EDYGA contains tyrosine (Tyr), which features a phenolic group capable of neutralizing free radicals through hydrogen atom transfer, thereby inhibiting lipid peroxidation. On the other hand, the terminal cysteine (Cys) in DLLCIC is a crucial amino acid for antioxidant reactions (28). The thiol group (–SH) can scavenge free radicals or form disulfide bonds with other thiol groups (29), thus providing antioxidant protection. Therefore, both peptides exhibit strong antioxidant potential. In this study, we aim to assess the antiglycation properties of EDYGA and DLLCIC using the BSA/Glucose model and explore the potential mechanisms behind these effects. The findings provide further evidence that certain antioxidant peptides can prevent the formation of AGEs.

## 2 Materials and methods

### 2.1 Chemicals and materials

Previous studies have obtained EDYGA (Glu-Asp-Tyr-Gly-Ala) and DLLCIC (Asp-Leu-Leu-Cys-Ile-Val) from *Pelodiscus sinensis* protein (27). In this study, peptides were synthesized using a solid phase technique, as described previously (27). Purities of all acquired peptides were > 98%. Glucose was purchased from Pricella Biotechnology (Wuhan, China), RPMI-1640 medium and bovine serum albumin (BSA) were obtained from Cellmax Technologies (Beijing, China), Sodium azide and DMSO were purchased from China National Pharmaceutical Group Chemical Reagents (Beijing, China), 0.25% trypsin–EDTA were purchased from Cienry Technologies (Huzhou, China) and phosphate buffer saline (PBS) were purchased from Genom Bio (Hangzhou, China), Carnosine was purchased from Sigma-Aldrich (St. Louis, MO, USA). CCK-8, BCA, ROS, SOD, GSH, MAD assay Kits and penicillin–streptomycin (100×) were purchased from Beyotime Laboratories (Shanghai, China).

### 2.2 Building of BSA/glucose glycation model

The method from Ou et al. (30) was used with minor modifications to determine the inhibitory effect of peptides on AGEs formation. In brief, BSA (20 mg/ml) and glucose (80 mmol/L) were dissolved in 0.2 M PBS (pH 7.4), Sodium azide (0.02%) was added to prevent bacterial growth, and the mixtures were incubated at 37°C for 3, 7, 14, 21, and 28 days.

The control groups were set up as follows: Group A without adding any samples; Group B without adding D-Glu and any samples; Group C without adding BSA and any samples; Group D without adding BSA; and Group E without adding D-Glu. There were five control groups in total. The experiment was conducted in triplicate.

### 2.3 Determination of NEG and AGEs products

#### 2.3.1 Effect of EDYGA and DLLCIC on NEG formation by UV–vis

Nonenzymatic glycosylation (NEG) analysis was performed as described by Wu et al. (31). BSA (20 mg/ml) and glucose (80 mmol/L) were dissolved in 0.2 M PBS (pH 7.4), followed by the addition of peptides (0.25, 0.50, 0.75, 1.00 mg/ml). Sodium azide (0.02%) was subsequently added, and the mixture was incubated at 37°C for 3, 7, 14, 21, and 28 days. The modified NEG levels were then assessed to evaluate the peptides’ inhibitory effects on NEG formation. The amount of NEG formed was quantified by measuring absorbance at 530 nm using a UV–visible spectrophotometer (UV-2000, UNICO Instruments Co., Ltd., China), as calculated using Equation 1.


(1) $${IR}_{NEG}=1-\frac{A_{\mathrm{Sample}}-A_{\mathrm{Contral}\;d}-A_{\mathrm{Contral}\;e}}{A_{\mathrm{Contral}\;a}-A_{\mathrm{Contral}\;b}-A_{\mathrm{Contral}\;c}}\times 100\%$$

#### 2.3.2 Effect of EDYGA and DLLCIC on AGEs formation measured by fluorescence spectrophotometry

To determine the direct effect of peptides on AGEs formation, an assay was performed as described by Ou et al. (30) with minor changes. BSA (20 mg/ml) and glucose (80 mmoL/L) were dissolved in 0.2 M PBS (pH 7.4), followed by the addition of peptides (0.25, 0.50, 0.75, and 1.00 mg/ml). Sodium azide (0.02%) was then added, and the mixture was incubated at 37°C for 3, 7, 14, 21, and 28 days, respectively. Thus, altered AGE levels were determined to assess the effects of peptides on the inhibition of AGEs formation. The number of AGEs formed was evaluated by measuring the fluorescence intensity at 370/440 nm (excitation/emission) using an F-7000 fluorescent photometer (Hitachi, Tokyo, Japan), as calculated using Equation 2.


(2) $${IR}_{\mathrm{AGEs}}=1-\frac{F_{\mathrm{Sample}}-F_{\mathrm{Contral}\;d}-F_{\mathrm{Contral}\;e}}{F_{\mathrm{Contral}\;a}-F_{\mathrm{Contral}\;b}-F_{\mathrm{Contral}\;c}}\times 100\%$$

### 2.4 FTIR analysis

FTIR spectroscopy was employed to examine structural alterations in glucose-modified protein at varying AGEs concentrations. After freeze-drying, the sample was mixed with dried KBr (1:100) in agate mortar and uniformly crushed into fragments with a spectral range of 4,000 ~ 400 cm^−1^. Scans were performed 32 times using a Perkin Elmer Spectrum 100 FTIR spectrometer (PerkinElmer, Inc., USA) (32).

### 2.5 Determination of glycated protein by SDS-PAGE

The experimental procedure described by Gu et al. (33) was followed with slight modifications. Under conditions where penicillin sodium and streptomycin sulfate were used to inhibit microbial growth, 20 mg/ml BSA was incubated with 80 μM glucose in the presence or absence of 1 mg/ml EDYGA or Carnosine in 0.2 M PBS (pH 7.4) at 37°C for 28 days. The influence of glucose on BSA modification and the inhibitory actions of EDYGA and carnosine were examined using SDS-PAGE, employing a 4% stacking gel and a 12% Separating gel. A sample volume of 2.5 μL was loaded into each lane, and the gels were subsequently stained with Coomassie Brilliant Blue R250. Collect the staining solution, add the decoloring solution (V methanol: V glacial acetic acid: V distilled water = 25: 10: 65), decolorize on the shaker overnight until the gel background color is removed, and the protein band is clearly recognizable. Electrophoresis was conducted with the Mini Gel III system (BioRad, Hercules, CA).

### 2.6 Molecular docking

The molecular docking simulations were conducted using MOE software, following Khan’s method with some modifications (34). The crystal structure of BSA (PDB ID: 4OR0) was obtained from the RCSB Protein Data Bank.^1^ Before docking, each structure was processed using the “QuickPrep” function in MOE software, which involved removing bound ligands and water molecules, adding hydrogen atoms, and performing energy minimization. The EDYGA and DLLCIC peptide sequences were then constructed and docked with the prepared BSA molecule, with each peptide undergoing 30 docking runs.

### 2.7 Cell culture

HUVEC cells were obtained from CAMS Cell Culture Center (Beijing, China), and cultured in endothelial cell medium (Sciencell, San Diego, California, USA) with 10% fetal bovine serum and 1% streptomycin–penicillin. The culture conditions contained a humidified atmosphere (95% air and 5% CO_2_ at 37°C), with a sterile water tray placed inside the incubator to provide the required humidity. Subculture the cells until they reach a state of normal growth.

### 2.8 Cell proliferation assay

Cell proliferation assay was performed using a CCK-8 agent according to the manufacturer’s protocol. After treatment, HUVEC cells (1,000 cells/well) were seeded into a 96-well plate and incubated overnight. Cells were treated with either vehicle (control) or different doses of peptides (1, 5, 10, 20, and 50 μM) 12 h before AGEs (500 μg/ml) stimulation and incubated for an additional 12 h. 10 μL of CCK-8 agent were added into the 96-well plate and incubated for 4 h at 37°C. Then, the plate was transferred into a microplate reader and the OD value was detected at 450 nm (Infinite E Plex, TECAN, Switzerland). Each test was established in three repetitions.

### 2.9 Statistical analysis

Data were expressed as the mean ± standard deviation (SD) of three replicated determinations and analyzed by SPSS 22 (SPSS Inc., Chicago, IL, USA). Data were analyzed using one-way analysis of variance (ANOVA). EC_50_ values were calculated using GraphPad Prism 5. A value of *p* < 0.05 was considered statistically significant. Graphs were drawn by Origin 19 (OriginLab Inc., Northampton, Massachusetts, USA).

## 3 Results and discussion

### 3.1 Effect of EDYGA and DLLCIC on NEG formation

Protein glycation generally consists of three reaction stages: early, intermediate, and late stage. In the early stage, carbonyl groups of reducing sugars react with free amino groups to form unstable early oxidation products. These Schiff bases further undergo rearrangement to form stable products-Amadori products (35). Nonenzymatic glycosylation (NEG) is an early glycosylation product formed by a complex series of non-enzymatic reactions between protein and glucose in the body.

**Figure 1:**
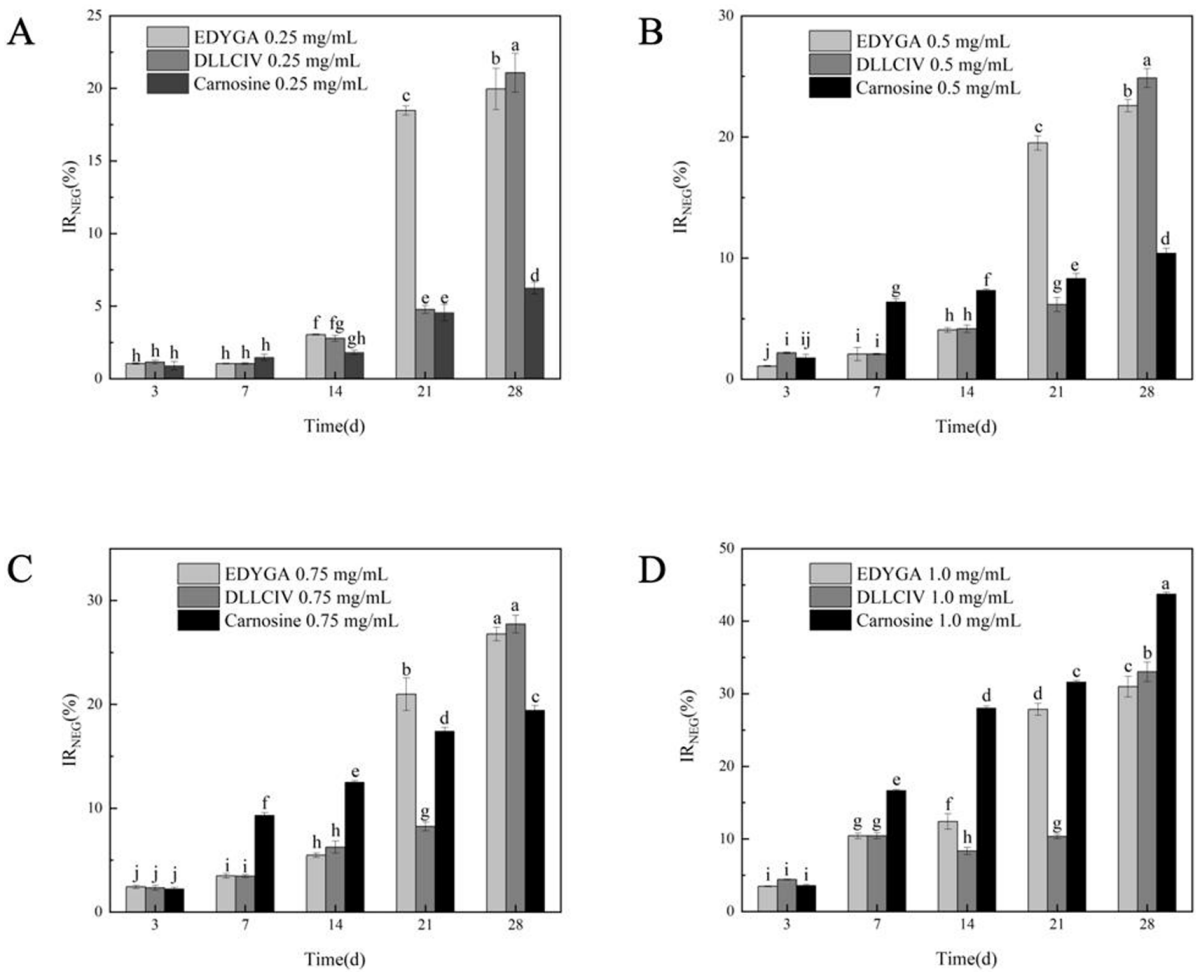
Effect of EDYGA, DLLCIC, and Carnosine on NEG formation in the BSA/glucose system. BSA (20 mg/mL), glucose (80 μM), and peptides (0.25, 0.50, 0.75, and 1.00 mg/mL) were incubated in PBS at 37°C in the dark for 3, 7, 14, 21, and 28 days. **(A)** Effect of 0.25 mg/mL peptides on NEG formation. **(B)** Effect of 0.50 mg/mL peptides. **(C)** Effect of 0.75 mg/mL peptides. **(D)** Effect of 1.00 mg/mL peptides. Significant differences (*p* < 0.05) are indicated by different letters.

The formation of NEG in the BSA/glucose system (3, 7, 14, 21, 28 days) treated with different concentrations of EDYGA and DLLCIC was evaluated. Figure 1 showed that EDYGA and DLLCIC inhibited the formation of NEG when added at concentrations ranging from 0.25 to 1.00 mg/ml. Carnosine is known to act as an antioxidant by inhibiting protein glycation, the production of metal chelates, and the accumulation of AGE during aging (36, 37). Similar to the positive control, carnosine, both EDYGA and DLLCIC exhibited an increased inhibitory rate of NEG formation with prolonged treatment time and higher concentration. After 28 days of incubation, 1.00 mg/ml of EDYGA and DLLCIC resulted in reductions in NEG formation of 30.98 and 33.03%, respectively, which are close to the 43.75% observed with the positive control (Figure 1D). This indicates that both EDYGA and DLLCIC exhibit significant inhibitory effects on NEG formation. Notably, at concentrations ranging from 0.25 to 0.75 mg/ml, the inhibitory rates of EDYGA and DLLCIC were higher than that of the positive control, carnosine, suggesting that both peptides exhibit more potent inhibition of NEG formation. In summary, the findings suggest that both EDYGA and DLLCIC significantly inhibit NEG formation, with increasing inhibition rates correlating with prolonged treatment duration and higher concentrations. Moreover, at lower concentrations, their inhibitory efficacy surpasses that of carnosine, with DLLCIC exhibiting a slightly higher inhibition rate than EDYGA.

### 3.2 Effect of EDYGA and DLLCIC on AGEs formation

**Figure 2:**
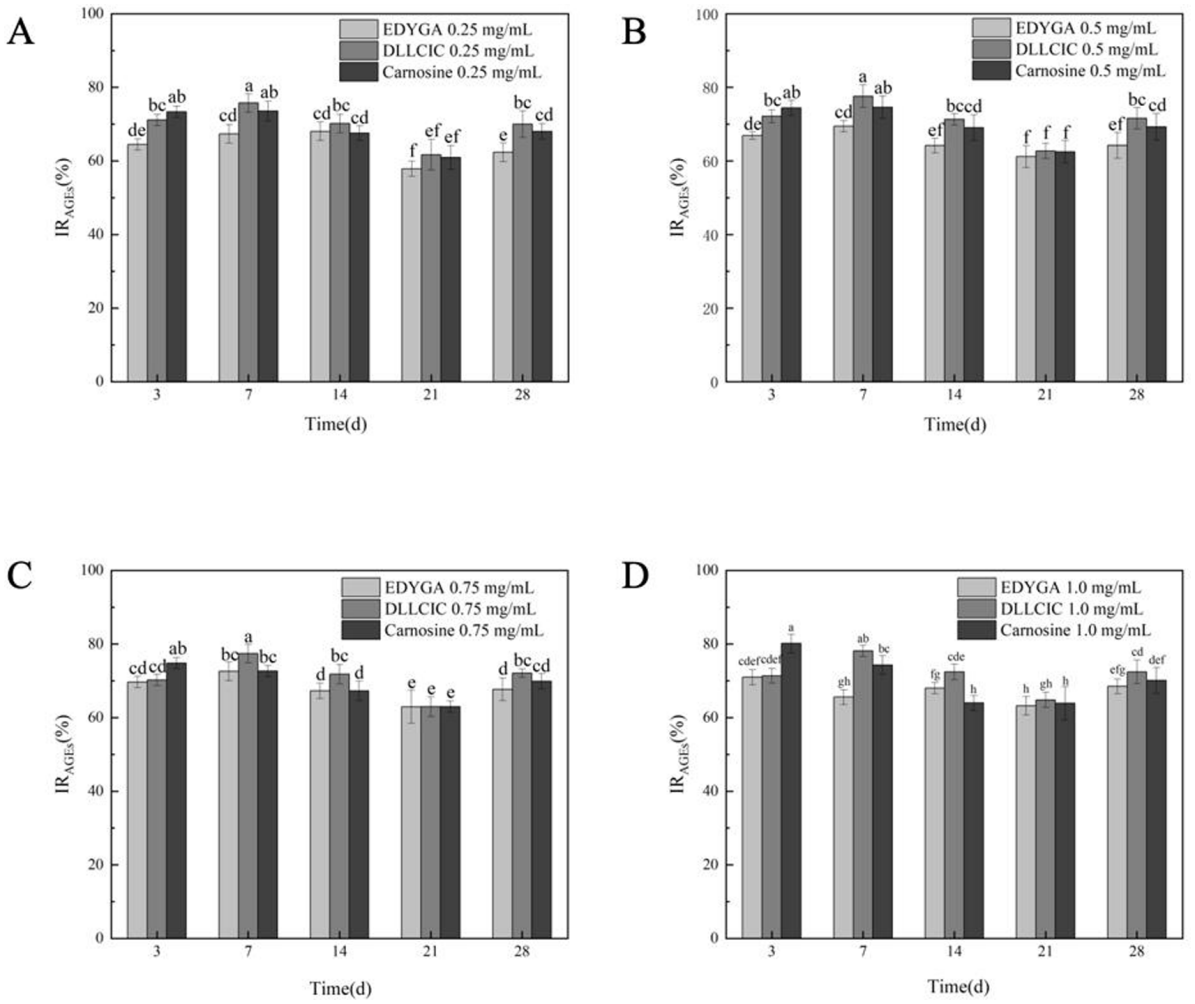
Effect of EDYGA, DLLCIC, and Carnosine on AGEs formation in the BSA/glucose system. BSA (20 mg/mL), glucose (80 μM), and peptides (0.25, 0.50, 0.75, and 1.00 mg/mL) were incubated in PBS at 37°C in the dark for 3, 7, 14, 21, and 28 days. **(A)** Effect of 0.25 mg/mL peptides on AGEs formation. **(B)** Effect of 0.50 mg/mL peptides. **(C)** Effect of 0.75 mg/mL peptides. **(D)** Effect of 1.00 mg/mL peptides. Significant differences (*p* < 0.05) are indicated by different letters.

AGEs were characterized based on their fluorescence properties. The formation of AGEs was assessed using a fluorescence spectrophotometer (38). The content of AGEs in each group was quantified by measuring the fluorescence intensity at the characteristic wavelength of AGEs. The data presented in Figure 2 indicate that the AGEs inhibitory rates of all three peptides at varying concentrations initially increase, followed by a decline, eventually stabilizing. This suggests that the inhibitory capacity of these peptides diminishes over prolonged treatment, possibly due to the development of some level of tolerance. At a concentration of 0.25 mg/ml, carnosine consistently outperforms EDYGA and DLLCIC in AGEs inhibition across all treatment durations. At 0.5 mg/ml, the inhibitory effects of the three peptides are comparable, with DLLCIC showing an inhibition rate equal to that of carnosine at days 7 and 14, indicating robust inhibitory efficacy. Although EDYGA shows a reduced inhibitory effect relative to DLLCIC, its inhibition remains statistically significant. At 1.0 mg/ml, the inhibitory effects of the *Pelodiscus sinensis* peptides approach or even exceed those of carnosine. The AGEs inhibitory rates of EDYGA and DLLCIC are closely associated with both treatment duration and concentration. Overall, these two peptides demonstrate the strongest inhibitory effects at higher concentrations and during the mid-term treatment period (14 days). While the inhibitory effects of the *Pelodiscus sinensis* peptides are inferior to carnosine at low concentrations, their efficacy gradually approaches or even exceeds that of carnosine at higher concentrations and longer treatment durations, particularly in the case of DLLCIC, which exhibits a sustained inhibitory effect. Wu and Yen (39) reported that rutin, quercetin, kaempferol, and epigallocatechin gallate (EGCG) exhibited inhibitory activities of 86.4, 79.5, 68.7, and 65.8%, respectively, after 7 days of incubation. These results are comparable to the maximum inhibitory activities of EDYGA and DLLCIC, which were 72.6 and 78.2%, respectively, after 7 days. This similarity suggests that EDYGA and DLLCIC are also effective in preventing high glucose-mediated protein modifications.

Our previous studies have shown that EDYGA and DLLCIC have strong antioxidant activity (27). Khanam et al. (34) found that the antioxidant activity of natural products is closely related to the antiglycative activity. Therefore, the inhibition effect on NEG and AGEs formation may be attributed to their antioxidant activity.

### 3.3 SDS-PAGE analysis

**Figure 3:**
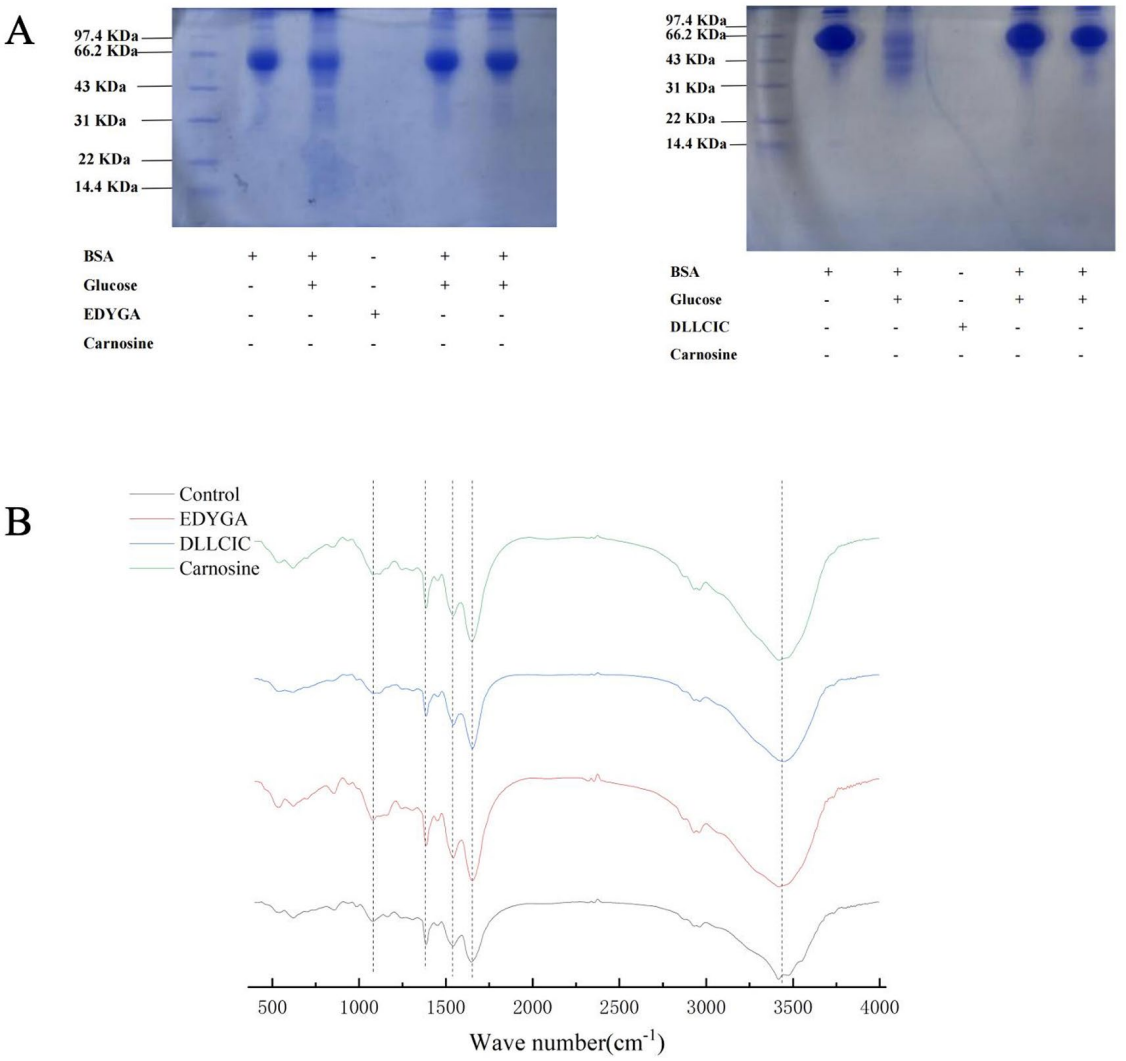
Effect of EDYGA and DLLCIC on AGEs formation in BSA/glucose system. BSA (20 mg/mL) and glucose (80 μM) and EDYGA (1.00 mg/mL) (or DLLCIC) were mixed in PBS and incubated in dark at 37°C for 28 days, respectively. **(A)** SDS-page **(B)** FTIR spectra.

BSA is a common protein widely used in glycation studies due to its abundance in plasma since it can be glycated at multiple sites (40). The inhibitory effects of EDYGA and DLLCIC on protein glycation were assessed using a BSA/glucose glycation model. As depicted in Figure 3A, SDS-PAGE analysis was conducted to examine BSA incubated with glucose, in the presence or absence of EDYGA or DLLCIC. The original BSA showed a distinct band at 66 kDa (Figure 3A, ‘lane 1’). This result is similar to the findings of Liu et al. (41). The non-glycated BSA band was observed around 70 kDa. However, BSA incubated with glucose without EDYGA or DLLCIC (Figure 3, ‘channel 2’) displayed a lighter color of the band because the active group of the BSA protein decreased after reacting with glucose. This causes the BSA molecule to bind less Coomassie Brilliant Blue, resulting in a lighter protein band after glycosylation. These results demonstrated that EDYGA or DLLCIC could inhibit AGEs generation *in vitro*.

Research has shown that EDYGA and DLLCIC contain electron-donating groups on the benzene ring (notably 3-methoxy and 4-hydroxyl), which can form a resonance-stabilized phenoxy radical, allowing them to function as natural antioxidants. The inhibitory effect of EDYGA and DLLCIC on AGEs formation is probably attributed to their capability of scavenging free radicals (27). It has been reported that the ability of antioxidants such as ferulic acid and resveratrol to inhibit AGEs has been studied using SDS-PAGE, yielding similar results (42, 43).

FTIR spectra of the BSA/glucose system incubated for 21 days with and without 1.00 mg/ml EDYGA (or DLLCIC) were analyzed in the range of 4,000–400 cm^−1^ (Figure 3B). In the main bands of proteins in the IR region, the amide A peak position occurs in the 3,400–3,300 cm^−1^ region (mainly N-H stretch), while the amide I peak position occurs in the 1,600–1,700 cm^−1^ region (mainly C=O stretch) (44, 45). The amide II band was located from 1,500 to 1,600 cm^−1^ (C-N stretch coupled with N-H bending mode) (7). The amide I peak mainly reflects the secondary structure of the protein. In the BSA/glucose system, band positions (amide I & II) of EDYGA (or DLLCIC)-treated groups were stronger than the control group, indicating more unaffected amide bonds and amino groups were present in the solution containing EDYGA (or DLLCIC).

It has been demonstrated that peaks at 1,000 cm^−1^ (O-H and C-C bonds) (46, 47) are enhanced, indicating that a glycosylation reaction between protein and reducing sugar occurred. Compared with the control group, the peak at 1,000 cm^−1^ is attenuated in EDYGA (or DLLCIC) treated groups. Thus, FTIR analysis of the BSA/glucose system with and without EDYGA (or DLLCIC) corroborates that EDYGA (or DLLCIC) plays an important role in maintaining the secondary structure of protein, and inhibiting the formation of AGEs. The result is similar to the findings of Wang et al. (48); myricetin and its derivatives protect the secondary structure of BSA by inducing binding with the protein, thereby preventing BSA glycation. Yang et al. (49) demonstrated as well that Citral inhibits AGE formation by competitively cross-linking with BSA.

### 3.4 Docking EDYGA and DLLCIC to BSA domain

**Figure 4:**
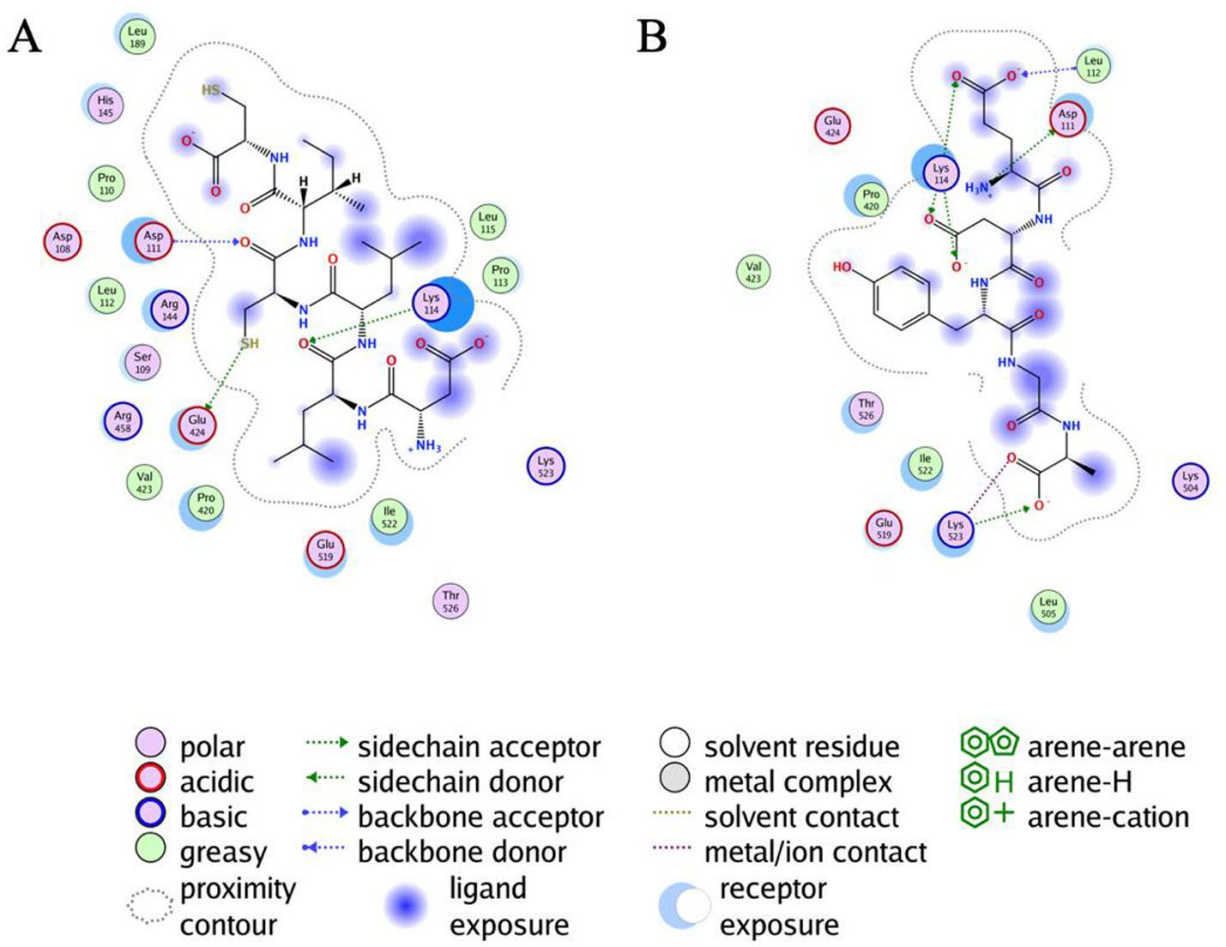
Results of the all atoms docking of DLLCIC and EDYGA with BSA protein. **(A)** Docking results of DLLCIC with the BSA molecule. **(B)** Docking results of EDYGA with the BSA molecule.

As shown in Table 1, DLLCIC interacts with BSA residues, such as Lys114, Asp111, and Glu424, primarily through hydrogen bonding. Hydrogen bonds are critical forces in molecular binding, indicating a high affinity between DLLCIC and BSA at the binding site. Additionally, there are hydrophobic interactions, for example, with Leu112 and Pro113, which contribute to the stable binding of DLLCIC to the protein (50, 51). These hydrophobic interactions not only enhance the ligand’s binding stability but also bolster its antioxidant capacity. Amino acid residues such as Lys114 and Glu424 are often found at the active sites of proteins and are closely linked to antioxidant reactions. The hydrogen bond formed by Lys114 may involve BSA’s active center, potentially impacting its function. The lysine residues have been shown to be hotspots for BSA glycation due to their high nucleophilic activity (52). The presence of a thiol group in DLLCIC, as suggested in the Figure 4, might interact with surrounding residues, which could help reduce the generation of free radicals, thereby enhancing antioxidant activity. Like many natural compounds, these molecules effectively protect the structural integrity of proteins and inhibit non-enzymatic glycation by competitively binding to proteins through hydrogen bonds or van der Waals forces (53). By binding to these residues, DLLCIC could indirectly enhance or modulate BSA’s antioxidant enzyme activity, thereby strengthening the antioxidant defense mechanisms.

**Table 1:** DLLCIC molecular docking ligand interactions report.

Receptor	Interaction	Distance	E (kcal/mol)
Glu424 (A)	H-donor	3.89	−0.7
Lys114 (A)	H-acceptor	2.96	−0.7
Asp111 (A)	H-acceptor	3.32	−1.0

As shown in Table 2, EDYGA interacts with multiple BSA residues, including Lys114, Asp111, Lys523, and Glu519, through hydrogen bonding. These hydrogen bonds likely stabilize the binding between EDYGA and BSA, indicating a high binding affinity for EDYGA. Similar to DLLCIC, EDYGA also engages in hydrophobic interactions with several residues within BSA, such as Leu112, Pro420, and Ile522. These hydrophobic interactions play a crucial role in stabilizing the ligand-protein complex, which may contribute to its antiglycation effects. While DLLCIC primarily binds to the central site of BSA, EDYGA forms interactions across a broader region, including with Lys523. This suggests that the two peptides may inhibit the formation of AGEs through different mechanisms. Both EDYGA and DLLCIC exhibit binding capabilities with BSA, and through stable hydrogen bonds and hydrophobic interactions, they may inhibit the formation of AGEs, thereby enhancing protection against glycosylation damage. The binding of DLLCIC to the core site exhibits stronger binding affinity, which may contribute to its enhanced activity.

**Table 2:** EDYGA molecular docking ligand interactions report.

Receptor	Interaction	Distance	E (kcal/mol)
Asp111 (A)	H-donor	2.99	−10.2
Lys114 (A)	H-acceptor	3.08	−0.5
Leu112 (A)	H-acceptor	3.11	−1.6
Lys523 (A)	H-acceptor	2.99	−4.4

### 3.5 Effect of EDYGA and DLLCIC on cytotoxicity of AGEs

**Figure 5:**
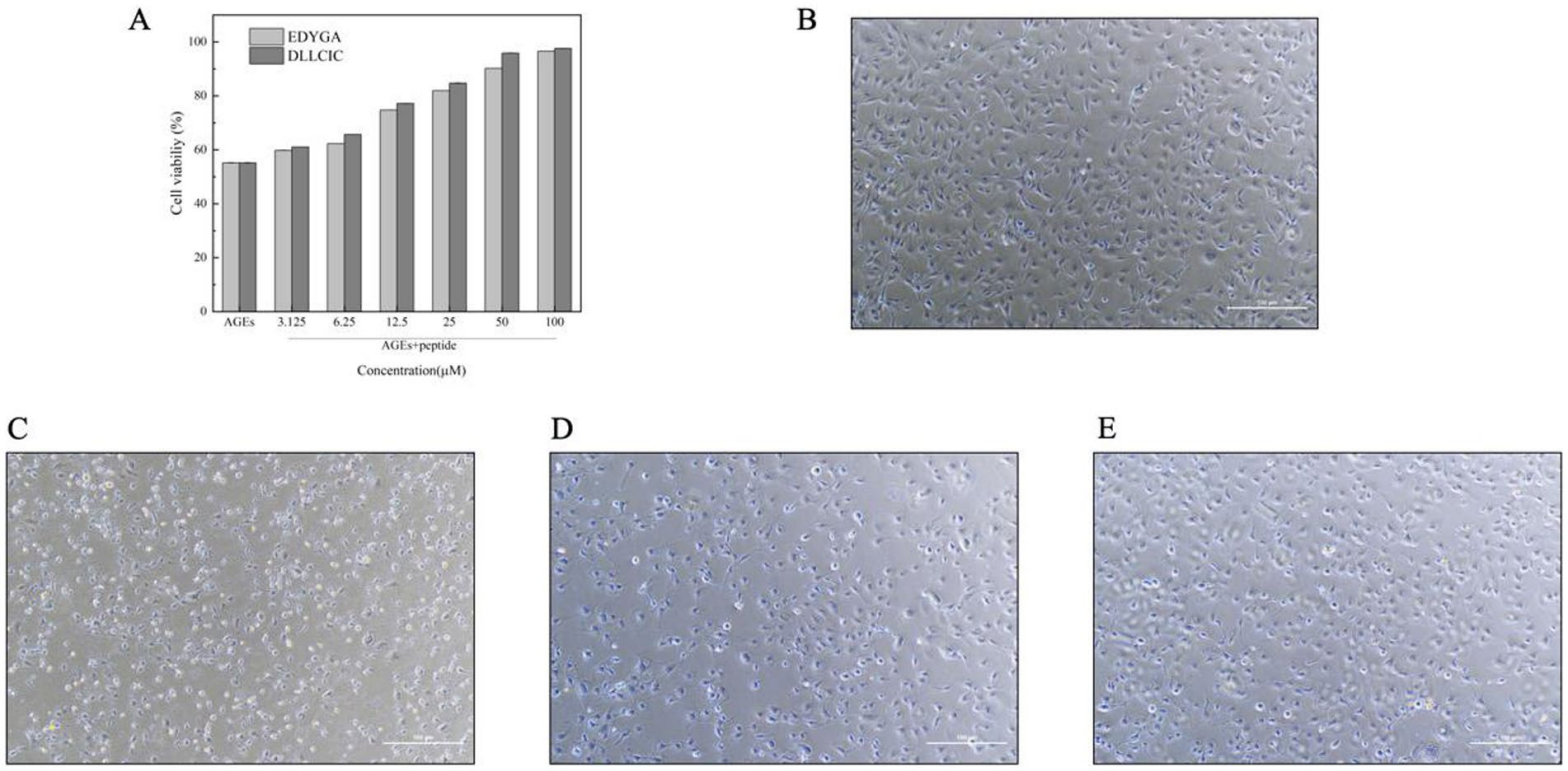
The Effect of EDYGA and DLLCIC on cytotoxicity of AGEs. **(A)** Cell viability assessed by CCK-8 method for EDYGA and DLLCIC’s recovery effects against AGEs damage. **(B)** Control group: cells cultured with medium for 24 h. **(C)** AGEs group: cells treated with AGEs (500 µg/mL) for 24 h. **(D)** EDYGA treatment group: cells treated with 20 µM EDYGA for 12 h, followed by AGEs (final concentration 500 µg/mL) for an additional 12 h. **(E)** DLLCIC treatment group: cells treated with 20 µM DLLCIC for 12 h, followed by AGEs (final concentration 500 µg/mL) for an additional 12 h.

As shown in Figure 5A, compared to the AGEs group, pretreatment with all concentrations of EDYGA and DLLCIC significantly (*p* < 0.01) increased the viability in AGEs-exposed cells. Miroliaei et al. (54) demonstrated that the treatment of four mammalian cell lines (peripheral blood mononuclear cells, human embryonic kidney cells—HEK293, normal human fibroblasts, and Chinese hamster ovary cells) with glycated proteins (AGEs) resulted in a significant decrease in cell viability, dropping from nearly 90% to a range of 37.13–41.35%. This finding confirms that AGEs can lead to a substantial reduction in cell viability. Using Origin software, we analyzed the cell viability of EDYGA and DLLCIC at different concentrations and calculated their half-maximal effective concentration (EC50) values. The EC_50_ for EDYGA was found to be 17.64 μM, while that for DLLCIC was 15.08 μM. For convenience, we selected concentrations of 20 μM for both EDYGA and DLLCIC to study their protective mechanisms.

Morphological changes in HUVECs cells treated with AGEs were examined by microscopy. The control group cells exhibited typical morphology with rounded nuclei and uniformly stained chromatin. However, after incubation with AGEs, the number of damaged cells increased significantly (Figure 5C). In the study by Gong et al. (55), HK-2 cells were treated with CML concentrations exceeding 0.8 mg/ml for 72 h. Over time, the cells gradually shifted from their normal elliptical and tightly packed arrangement to a spindle shape. Eventually, they transformed into myofibroblasts, leading to detachment and cell death. Pretreated with EDYGA and DLLCIC (20 μM) significantly (*p* < 0.01) decreased the number of damaged cells compared to AGEs damaged group. This suggests that both EDYGA and DLLCIC have inhibitory effects on the formation of AGEs, as indicated by the results shown in the Figures 5D,E. DLLCIC appearing to be more effective.

## 4 Conclusion

The study demonstrated that the peptides EDYGA and DLLCIC, derived from *Pelodiscus sinensis*, exhibit significant antiglycation activities by inhibiting the formation of NEG and AGEs. Both peptides showed comparable or even superior inhibitory effects compared to the well-known antiglycation agent, carnosine, particularly at higher concentrations and prolonged incubation times. The study also revealed that these peptides can maintain the secondary structure of proteins, as evidenced by FTIR analysis and SDS-PAGE, and enhance cell viability in AGEs-induced cytotoxic conditions. The molecular docking simulations provided insight into the mechanisms by which these peptides exert their effects, suggesting that the hydrogen bonding and hydrophobic interactions between the peptides and BSA residues play crucial roles in their antiglycation activity. Specifically, DLLCIC exhibited stronger binding to the central site of BSA, potentially contributing to its slightly higher efficacy compared to EDYGA. However, there are some limitations to this study. Firstly, the bioavailability and *in vivo* efficacy of these peptides remain unclear. Future studies should focus on their pharmacokinetics and therapeutic effects in animal models. Secondly, while the study suggests antioxidant properties, the exact molecular mechanisms of AGE inhibition by EDYGA and DLLCIC are not fully understood. Further research is needed to elucidate the pathways involved. Additionally, a broader range of peptide concentrations and longer treatment durations should be tested to optimize conditions for AGE inhibition.

Given the increasing incidence of glycation-related diseases, such as diabetes and its complications, the findings of this study offer promising potential for the development of EDYGA and DLLCIC as natural antiglycation agents. Future research should focus on in vivo studies to further validate these findings and explore the clinical applicability of these peptides. Additionally, investigations into the synergistic effects of these peptides with other natural compounds could pave the way for the development of more effective antiglycation therapies.

## Data Availability

The original contributions presented in the study are included in the article/supplementary material, further inquiries can be directed to the corresponding author.

## Footnotes

^1^ http://www.rcsb.org/
