# Corrigendum: Inhibition effect of AGEs formation *in vitro* by the two novel peptides EDYGA and DLLCIC derived from *Pelodiscus sinensis*

**DOI:** 10.3389/fnut.2025.1571244

**Published:** 2025-02-26

**Authors:** Nuo Chen, Nan Wang, Qiaoyun Fang, Zuolong Yu, Yiyuan Hu, Jiancang Jin, Shengli Yang

**Affiliations:** ^1^The College of Pharmaceutical Science, Zhejiang University of Technology, Hangzhou, China; ^2^College of Biology and Environmental Engineering, Zhejiang Shuren University, Hangzhou, China

**Keywords:** peptides, inhibition, AGEs, molecular docking, antiglycation

In the published article, there was an error in the **Funding statement**. The incorrect statement read as “The author(s) declare that financial support was received for the research, authorship, and/or publication of this article. This work was supported by the Basic Public Welfare Research Program of Zhejiang Province (No. LGN22C200024).” The correct **Funding statement** appears below.

“The author(s) declare that financial support was received for the research, authorship, and/or publication of this article. This work was supported by the Basic Public Welfare Research Program of Zhejiang Province (No. LGN22C200024). A key project funded by the Science and Technology Department of Zhejiang Province (No. 2023C02042, 2018C02017).”

The authors apologize for this error and state that this does not change the scientific conclusions of the article in any way. The original article has been updated.

